# The Surgeon’s Proficiency Affected Survival Outcomes of Minimally Invasive Surgery for Early-Stage Cervical Cancer: A Retrospective Study of 851 Patients

**DOI:** 10.3389/fonc.2021.787198

**Published:** 2021-11-16

**Authors:** Ying Yang, Yue Huang, Zhengyu Li

**Affiliations:** ^1^ Department of Gynecology and Obstetrics, West China Second University Hospital, Sichuan University, Chengdu, China; ^2^ Key Laboratory of Pediatric Diseases and Birth Defects of Ministry of Education, West China Second University Hospital, Sichuan University, Chengdu, China

**Keywords:** early-stage cervical cancer, minimally invasive surgery, open surgery, surgeon’s proficiency, survival outcomes

## Abstract

**Purpose:**

To compare the clinical outcomes of patients with early-stage cervical cancer who underwent minimally invasive surgery (MIS) by surgeons in different phases and evaluate whether the proficiency of surgeons affects the survival outcomes.

**Materials and Methods:**

A total of 851 patients with early-stage cervical cancer who underwent radical hysterectomy between January 2008 and June 2018 (every year from January to June) at a tertiary hospital were retrospectively analyzed. We categorized patients into four phases according to their sequence (phase one, 1-10 cases; phase two: 11-20 cases; phase three: 21-30 cases; phase four: > 30 cases). Demographics and clinical and pathological data were collected and analyzed.

**Results:**

There were no statistical differences between the open surgery and MIS groups regarding three- and five-year overall survival (OS) and disease-free survival (DFS). The OS and DFS of patients in the MIS group in phase one were significantly lower than those in later phases and those in the open surgery group after adjustment (OS, *P* = 0.009; HR, 2.896; 95%CI, 1.303-6.435; DFS, *P* = 0.009; HR, 2.712; 95%CI, 1.289-5.706). Survival outcomes were not statistically significant when comparing different surgeons.

**Conclusion:**

The phase one cases of MIS had lower OS and DFS than those in later phases and those in the open surgery group. Thus, we suggest that the proficiency of surgeons is associated with survival outcomes of MIS. Favorable outcomes can be obtained after a certain number of MIS cases.

## Introduction

According to statistics from the International Agency for Research on Cancer, the incidence of cervical cancer was 604,000 in 2020, making it the fourth most common gynecological cancer worldwide (1). Radical hysterectomy (RH) with lymphadenectomy remains one of the preferred treatments for patients with cervical cancer diagnosed at the International Federation of Gynecology and Obstetrics (FIGO) stage IA-IIA (2). In recent decades, minimally invasive surgery (MIS) has become more common, displacing the use of traditional open surgery for early-stage cancer. However, the Laparoscopic Approach to Cervical Cancer (LACC) trial observed that disease-free survival (DFS) at 4.5 years and overall survival (OS) at three years of MIS were significantly lower than those of open surgery among women with stage IA1-IB1 cervical cancer, which remains controversial (3). Potential explanations include increasing tumor spillage due to the application of a uterine manipulator, the effect of CO_2_ insufflation, the volume of surgery, and the surgeon’s proficiency (4–7). Several studies have focused on the correlation between surgeon proficiency and clinical outcomes in recent years. Lan Ying Li et al. found that compared to open surgery, more cases were required for surgeons performing minimally invasive RH to reach an acceptable five-year DFS (8). Kim et al. demonstrated that surgeons’ proficiency in the MIS group significantly affected progression-free survival (PFS) (9). Liu et al. concluded that the learning curve could be a probable reason for poor outcomes of MIS by analyzing stage IB cervical cancer patients treated with RH by one surgeon for 15 years (10). Nevertheless, existing studies have limitations such as low sample sizes, incomplete follow-up information, and the absence of clinicopathological features of patients. There is still insufficient evidence regarding whether a surgeon’s proficiency is associated with survival outcomes in MIS. We aimed to explore the effects of surgeons’ proficiency in MIS on short- and long-term clinical outcomes and whether it accounts for the clinical outcomes of MIS.

## Materials and Methods

### Study Design and Participants

This study was approved by the Institutional Ethics Committee of West China Second University Hospital, and all participants provided their written informed consent to participate in this study. Cervical cancer patients with consecutive FIGO (2009) stage IA-IIA treated with RH between January 2008 and June 2018 (every year from January to June) were retrospectively analyzed. The inclusion criteria were: (1) patients with FIGO stage IA1 with lymphovascular space invasion (LVSI), IA2, IB, and selected IIA cervical cancer; (2) patients who underwent standard surgical treatment, which was performed by five specific surgeons according to the National Comprehensive Cancer Network guidelines, a modified RH (Type B of the Querleu and Morrow (Q-M) surgical classification system) with pelvic lymphadenectomy (PLND) in stage IA1 with LVSI and stage IA2, and an RH (Type C of the Q-M surgical classification system) with PLND with/without para-aortic lymphadenectomy in stage IB to IIA (2, 11, 12). (3) Patients with histological subtypes of squamous cell carcinoma, adenocarcinoma, or adenosquamous carcinoma, regardless of histological grading. The exclusion criteria were as follows: (1) patients with incomplete data or complete loss to follow-up; (2) patients with severe fundamental diseases such as immune deficiency or other malignant tumors; and (3) patients with cervical cancer in pregnancy.

Complete information, including demographics and clinical and pathological information, was extracted from the hospital information system by two investigators (Y.Y. and Y.H.). The demographics extracted included age, menstruation (menopause or not), and body mass index (BMI). The clinical information extracted included diagnosis, FIGO (2009) stage, surgical approach, surgeon name, date of surgery, hospital stay, duration of surgery, estimated blood loss, CO_2_ pneumoperitoneum in MIS, number of resected lymph nodes, pre- and post-operative treatment, and imaging data. Pathological information included histologic subtype, grading, LVSI, stromal invasion depth, parametrial involvement, vaginal margin involvement, and lymph node metastasis. Recurrence was confirmed by clinical findings, radiological examinations, and pathology reports. OS and DFS were the primary outcomes of this study. OS was defined as the time interval between the date of surgery and the date of death. DFS was defined as the time interval between the date of surgery and either the date of the first recurrence or death.

### Statistical Analysis

The data were analyzed using the Statistical Package for the Social Sciences software version 22.0 (IBM Corp, Armonk, NY, USA). Missing values were statistically imputed by multiple imputations using logistic regression and predictive mean matching (13). The enumeration data were analyzed using the chi-square test. The measurement data were analyzed *via* t-test and Mann-Whitney U test between two groups, while an analysis of variance and Kruskal-Wallis H test were used to compare multiple groups. Survival curves were generated using the Kaplan-Meier method and analyzed using the log-rank test and multivariable Cox proportional hazards regression models. All the *P*-values reported are two-sided. A *P*-value of < 0.05 was considered statistically significant.

## Results

### Study Population

A total of 851 patients were included in the study: 510 in the open surgery group and 341 in the MIS group (no robotic RH). Their general characteristics are summarized in **Table 1**. The mean age of the patients was 46.51 years (range 18-73 years; standard deviation [SD] 9.33), and most patients (471, 55.35%) were diagnosed with stage IB1. There were no significant differences between the open surgery and MIS groups in terms of age, menopause, BMI, histologic subtype, and grading. Advanced FIGO stage and deep cervical stromal invasion were more frequent in the open surgery group. The median length of hospital stay was eight days (range 3-30) in the open surgery group and seven days (range 3-21) in the MIS group (*P* < 0.001). The median duration of surgery was 200 minutes (range 85-510) in the open surgery group and 240 minutes (range 75-450) in the MIS group (*P* < 0.001), and the median volume of blood loss was 400 mL (range, 50-2500 mL) in the open surgery group and 200 mL (range, 10-4500 mL) in the MIS group (*P* < 0.001).

**Table 1:** General characteristics of the study population stratified by surgical approach.

Characteristic	Open surgery (n=510, %)	Minimally invasive surgery (n=341, %)	*P* value
Age (year) (mean ± SD)	46.70 ± 9.39	46.22 ± 9.25	0.456
Menopause	170 (33.3)	114 (33.4)	0.976
BMI (kg/m2) (mean ± SD)	22.58 ± 2.80	23.06 ± 4.36	0.074
Histologic subtype			0.297
squamous-cell carcinoma	417 (81.8)	284 (83.3)	
adenocarcinoma	65 (12.7)	46 (13.5)	
adenosquamous carcinoma	28 (5.5)	11 (3.2)	
FIGO stage			<0.001
IA	24 (4.7)	17 (5.0)	
IB1	234 (45.9)	237 (69.5)	
IB2-IIA	252 (49.4)	87 (25.5)	
Histologic grading			0.149
G1	21 (4.1)	17 (5.0)	
G2	62 (12.2)	42 (12.3)	
G3	378 (74.1)	233 (68.3)	
Gx	49 (9.6)	49 (14.4)	
Stromal invasion depth			<0.001
<1/2	203 (39.8)	170 (49.9)	
≥1/2	307 (60.2)	171 (50.1)	
LVSI	308 (60.4)	208 (61.0)	0.860
Parametrial invasion	63 (12.4)	43 (12.6)	0.911
Positive vaginal margin	92 (18.0)	45 (13.2)	0.060
Lymph node metastasis	99 (19.4)	73 (21.4)	0.477
Hospital stay, median (range, days)	8 (3-30)	7 (3-21)	<0.001
Duration of surgery, median (range, min)	200 (85-510)	240 (75-450)	<0.001
Estimated blood loss, median (range, ml)	400 (50-2500)	200 (10-4500)	<0.001
Postoperative adjuvant treatment	399 (78.2)	285 (83.6)	0.054

BMI, body-mass index; FIGO, International Federation of Gynecology and Obstetrics; LVSI, lymph-vascular space invasion.

### Clinical Outcomes in Different Surgical Approaches

**Figure 1:**
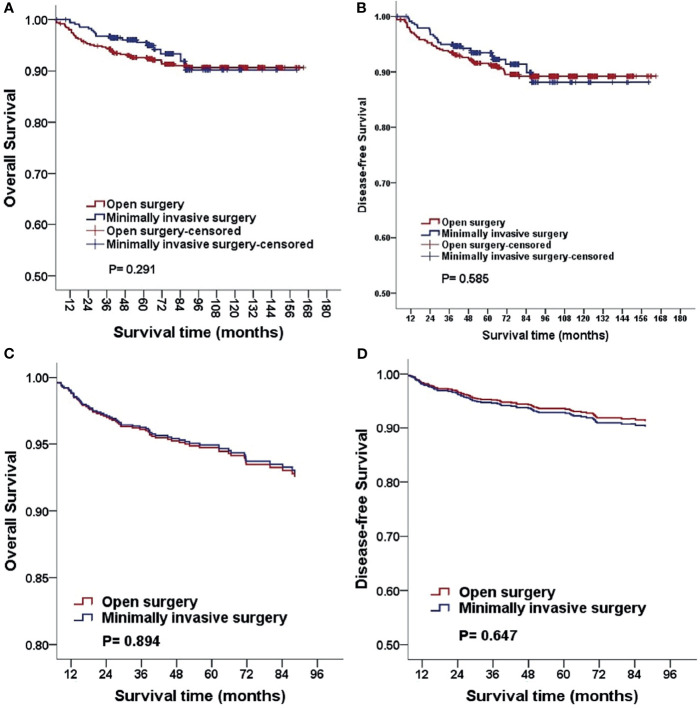
Kaplan-Meier analysis of the OS and DFS for patients stratified by surgical approach **(A, B)**. The OS and DFS adjusted for clinicopathological factors for patients stratified by surgical approach **(C, D)**. MIS, minimally invasive surgery.

The median follow-up duration was 88.6 and 62.2 months in the open surgery and MIS groups, respectively. With regard to the three- and five-year OS and DFS, statistically significant differences were not observed (three-year OS, 94.5% *vs*. 96.4%; five-year OS, 93.4% *vs*. 96.0%; *P* = 0.291; three-year DFS: 94.4% *vs*. 94.6%, five-year DFS: 92.8% *vs*. 94.2%, *P* = 0.585). After adjusting for age, BMI, FIGO stage, histologic subtype, and grading, identical results were obtained using multivariable Cox proportional hazards regression models [OS: *P* = 0.894, HR 1.039, 95%CI (0.593, 1.820); DFS, *P* = 0.647; HR, 0.891; 95%CI (0.544, 1.460); **Figure 1**]. In addition, we analyzed the survival outcomes of the open surgery and MIS groups for every surgeon and found that the survival outcomes were still not statistically significant (all *p-*values > 0.05).

### Survival Outcomes in Different Phases and Surgeons

**Figure 2:**
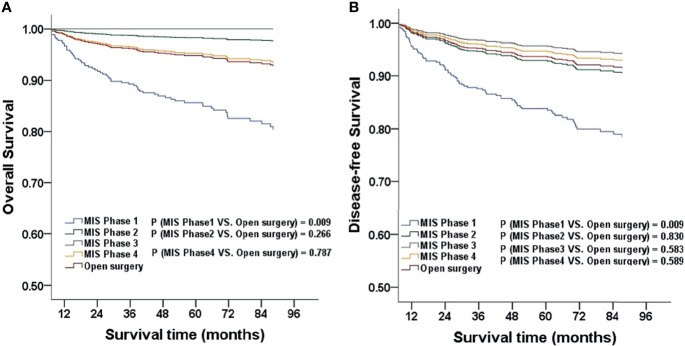
The OS and DFS adjusted for clinicopathological factors for patients stratified by phase **(A, B)**. MIS, minimally invasive surgery.

For every surgeon, we categorized patients who underwent surgery into four phases according to their sequence (phase one, 1-10 cases; phase two, 11-20 cases; phase three, 21-30 cases; phase four, more than 30 cases). Considering open surgery as an advanced technique, we did not categorize patients in this group into different phases. When stratified by surgical phases, the OS and DFS of the MIS group in phase one were significantly lower than those in later phases and in the open surgery group after adjusting for age, BMI, FIGO stage, histologic subtype, and grading. The three-year OS was 91.8% (45/49), and the five-year OS was 87.8% (43/49) in phase one of the MIS group (*P* = 0.009; HR, 2.896; 95%CI, 1.303-6.435). The three-year DFS was 91.8% (45/49) and the five-year DFS was 85.7% (42/49) in phase one of the MIS group (*P* = 0.009; HR, 2.712; 95%CI, 1.289-5.706; **Figure 2**). We obtained similar results when analyzing the surgeons separately (**Table 2**). Statistical differences were observed in the median number of resected lymph nodes (*p* < 0.001). There were no significant differences among the four phases with respect to perioperative characteristics, including hospital stay, duration of surgery, volume of blood loss, and volume of CO_2_ pneumoperitoneum after adjustment (**Table 3**).

**Table 2:** Survival outcomes of different surgeons in different phases in minimally invasive surgery.

Variable		Overall survival				Disease-free survival			
		3-year (%)	5-year (%)	HR (95% CI)	*P* value	3-year (%)	5-year (%)	HR (95% CI)	*P* value
Open surgery		94.3	92.4	1 (ref.)	–	93.4	91.3	1 (ref.)	–
Surgeon A	P1	100	90	1.834 (0.237-14.17)	0.561	90	80	3.464 (0.792-15.162)	0.099
	P2	100	100	–	–	100	100	–	–
	P3	100	100	–	–	100	100	–	–
	P4	100	100	–	–	100	100	–	–
Surgeon B	P1	100	100	–	–	100	100	–	–
	P2	100	100	–	–	100	90	1.567 (0.199-12.341)	0.670
	P3	100	100	–	–	90	90	2.627 (0.336-20.542)	0.357
	P4	100	100	–	–	100	100	–	–
Surgeon C	P1	90	80	2.595 (0.732-9.197)	0.140	90	80	2.397 (0.698-8.226)	0.165
	P2	100	100	–	–	88.9	88.9	1.368 (0.183-10.231)	0.760
	P3	100	100	–	–	88.9	88.9	1.501 (0.202-11.126)	0.691
	P4	97.9	93.4	0.742 (0.263-2.094)	0.573	96.8	93.7	0.617 (0.220-1.725)	0.357
Surgeon D	P1	77.8	77.8	5.866 (1.301-26.441)	**0.021**	77.8	77.8	4.696 (1.061-20.776)	**0.042**
	P2	100	90	1.273 (0.164-9.901)	0.817	90	90	1.261 (0.165-9.662)	0.823
	P3	100	100	–	–	100	100	–	–
	P4	91.8	91.8	1.420 (0.585-3.446)	0.439	91.7	89.3	1.407 (0.621-3.187)	0.414
Surgeon E	P1	90	78.8	5.020 (1.106-22.790)	**0.037**	90	78.8	4.256 (0.951-19.037)	0.058
	P2	100	100	–	–	100	90	1.866 (0.247-14.102)	0.546
	P3	100	100	–	–	100	100	–	–
	P4	100	100	–	–	100	100	–	–
Total	P1	91.8	87.8	2.896 (1.303-6.435)	**0.009**	91.8	85.7	2.712 (1.289-5.706)	**0.009**
	P2	100	96.7	0.321 (0.043-2.381)	0.266	93.9	91.6	1.122 (0.392-3.213)	0.830
	P3	100	100	–	–	94.9	94.9	0.671 (0.162-2.782)	0.583
	P4	96	92.1	0.907 (0.446-1.843)	0.787	95.5	91.6	0.831 (0.426-1.624)	0.589

P values < 0.05 are in bold. P, phase; HR, hazard ratio; CI, confidence interval; ref, reference.

**Table 3:** Perioperative outcomes in different phases in minimally invasive surgery.

Characteristic	Minimally invasive surgery				
	Phase 1	Phase 2	Phase 3	Phase 4	*P* value
Hospital stay (days) (mean ± SD)	7.88 ± 2.88	7.32 ± 2.33	7.50 ± 2.39	7.15 ± 2.14	0.235
Duration of surgery (min) (mean ± SD)	252.73 ± 50.85	25.72 ± 56.88	238.89 ± 45.39	245.37 ± 62.08	0.659
Estimated blood loss, median (range, ml)	175 (20-800)	200 (40-600)	200 (80-4500)	200 (10-1000)	0.479
CO2 pneumoperitoneum, median (range, L)	590 (80-1200)	483.5 (160-1090)	669 (255-1200)	684 (100-1300)	0.123
No. of lymph nodes resected, median (range)	18.5 (4-33)	19 (3-57)	17.5 (6-50)	23 (2-53)	<0.001

SD, standard deviation.

**Figure 3:**
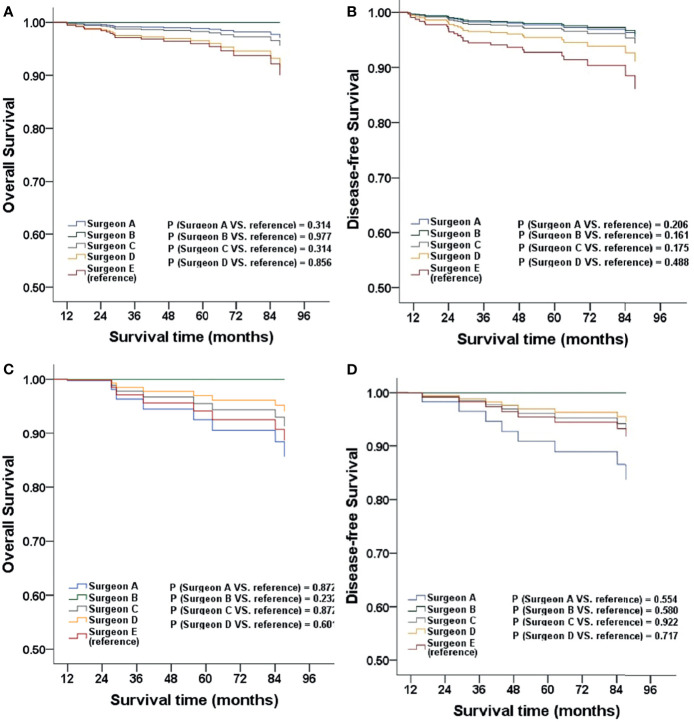
The OS and DFS adjusted for clinicopathological factors for patients stratified by surgeon **(A, B)**, patients stratified by surgeon in MIS phase one **(C, D)**.

When comparing different surgeons, the survival outcomes were not statistically significant after adjustment. Furthermore, the subgroup analysis in the corresponding phases showed the same result (all *P* > 0.05; **Figure 3**).

## Discussion

The World Health Organization proposed that patients with cervical cancer must be managed appropriately to accelerate the elimination of the disease, prompting more attention to the treatment of cervical cancer in recent years. Previous studies demonstrated that MIS and open surgery had comparable oncological outcomes (14–16). In contrast, several recent studies showed poorer oncological outcomes, especially lower DFS in patients treated with MIS (3, 17–19). As the findings were conflicting, the superiority or inferiority of MIS remains unclear. In our study, there were no statistical differences in the MIS and open surgery groups with regard to three- and five-year OS and DFS, and the subgroup analysis performed by different surgeons reached the same conclusion. However, when we compared different phases in MIS with open surgery, the OS and DFS of phase one were in line with those of the LACC trial. The perioperative outcomes such as hospital stay and blood loss were significantly better in the MIS group, whereas the duration of surgery was significantly shorter in the open surgery group, which is in accordance with many other studies.

According to the NCCN guideline (2), the standard approach for RH is the open surgery, and the oncologic risks of MIS should be informed carefully to patients given the findings of poorer survival outcomes of MIS recently. The development of MIS was affected by relevant research works. Therefore, it is essential to clear the reasonable application of MIS to achieve maximum benefits for patients. Pedestrian et al. showed that patients with tumor dimension less than two centimeters still suitable for MIS, but more studies are needed to refine on criteria (20).

Several potential reasons may explain the limitations of MIS, such as the use of a uterine manipulator, insufflation gas, intracorporeal colpotomy under CO_2_ pneumoperitoneum, and the proficiency of the surgeon (7, 21–23). Gynecologic oncologists have designed innovative techniques and devices to reduce the potential negative consequences of these risks in recent years. For example, Hiroyuki Kanao et al. devised the “no-look, no-touch” technique for preventing intraoperative tumor spillage (24). Moreover, Peng Yuan et al. performed abdominal uterine manipulation and enclosed colpotomy (23). However, prospective studies are needed to evaluate the effects of these surgical techniques on oncological outcomes.

Previous studies have examined the learning curve in terms of some clinical parameters, such as the number of cases needed to reach a stable operation duration or to obtain a relatively low hemorrhage volume (25). Survival outcomes have rarely been reported. A recent study by Kim et al. concluded that improvement of surgical performance could be achieved after 13 cases of MIS (9). Pedestrian et al. demonstrated that the peak of reduction of the recurrence risk was the 19^th^ MIS (26). Sert et al. showed that there was a higher number of recurrences in the first 50 cases (27). In this regard, our findings were in line with the results of previous studies that the first ten cases were significantly inferior to subsequent cases. Moreover, favorable survival outcomes observed after the first ten cases also demonstrated the effect of surgeon proficiency. There was no statistical significance when comparing the different surgeons, indicating that favorable outcomes can be obtained by practicing the MIS technique after a certain number of cases. Based on our findings, we offer the following suggestions. First, surgeons should undergo standardized training to improve their surgical skills (28). Second, surgeons performing MIS should have some standard qualification. For example, surgeons can only perform laparoscopic surgery after completing a certain number of virtual surgeries through surgery simulators. Third, beginners should be overseen by experienced surgeons, particularly for the first dozen or so cases.

The main strength of this study is the large sample size. In addition, compared with other studies, we analyzed more clinical parameters, making our results more robust and applicable to a larger population. However, our study has several limitations. Due to its retrospective nature, there could be bias in the patient selection. Furthermore, we did not evaluate the effect of different surgical techniques and devices in different phases. More studies on specific surgical approaches and techniques, including conventional multiport laparoscopic and laparo-endoscopic single-site surgery with/without technological innovations, are needed. Although there was no robotic case in our study, some relevant studies showed that robotic and laparoscopic approaches were similar in perioperative and postoperative outcomes (29), future studies could consider this aspect.

In conclusion, our retrospective study demonstrated that phase one cases of MIS had lower OS and DFS than those in later phases or that underwent open surgery. Thus, we suggest that the proficiency of the operating surgeon is associated with the survival outcomes of MIS. More favorable outcomes can be obtained after a certain number of MIS cases have been performed.

## Data Availability Statement

The original contributions presented in the study are included in the article/supplementary material. Further inquiries can be directed to the corresponding author.

## Ethics Statement

The studies involving human participants were reviewed and approved by the Institutional Ethics Committee of West China Second University Hospital. The patients/participants provided their written informed consent to participate in this study.

## Author Contributions

YY and ZYL contributed to data collection and study design. YY and YH contributed to data analysis. All authors contributed to the article and approved the submitted version.

## Funding

This study was supported by grants from the Sichuan Youth Foundation of Science of Technology (Grant number: 2015JQ0026).

## Conflict of Interest

The authors declare that the research was conducted in the absence of any commercial or financial relationships that could be construed as a potential conflict of interest.

## Publisher’s Note

All claims expressed in this article are solely those of the authors and do not necessarily represent those of their affiliated organizations, or those of the publisher, the editors and the reviewers. Any product that may be evaluated in this article, or claim that may be made by its manufacturer, is not guaranteed or endorsed by the publisher.
